# Contrasting impacts of ocean acidification and warming on the molecular responses of CO_2_-resilient oysters

**DOI:** 10.1186/s12864-017-3818-z

**Published:** 2017-06-02

**Authors:** Priscila Goncalves, Emma L. Thompson, David A. Raftos

**Affiliations:** 1 Department of Biological Sciences, Macquarie University, Sydney, NSW 2109 Australia; 2 Sydney Institute of Marine Science, Chowder Bay, Sydney, NSW 2088 Australia; 3 Present Address: School of Life and Environmental Science, The University of Sydney, Sydney, NSW 2006 Australia

**Keywords:** Climate change, Gene expression, Proteomics, *Saccostrea glomerata*, Selective breeding, Thermal stress, Low pH

## Abstract

**Background:**

This study characterises the molecular processes altered by both elevated CO_2_ and increasing temperature in oysters. Differences in resilience of marine organisms against the environmental stressors associated with climate change will have significant implications for the sustainability of coastal ecosystems worldwide. Some evidence suggests that climate change resilience can differ between populations within a species. B2 oysters represent a unique genetic resource because of their capacity to better withstand the impacts of elevated CO_2_ at the physiological level, compared to non-selected oysters from the same species (*Saccostrea glomerata*). Here, we used proteomic and transcriptomic analysis of gill tissue to evaluate whether the differential response of B2 oysters to elevated CO_2_ also extends to increased temperature.

**Results:**

Substantial and distinctive effects on protein concentrations and gene expression were evident among B2 oysters responding to elevated CO_2_ or elevated temperature. The combination of both stressors also altered oyster gill proteomes and gene expression. However, the impacts of elevated CO_2_ and temperature were not additive or synergistic, and may be antagonistic.

**Conclusions:**

The data suggest that the simultaneous exposure of CO_2_-resilient oysters to near-future projected ocean pH and temperature results in complex changes in molecular processes in order to prevent stress-induced cellular damage. The differential response of B2 oysters to the combined stressors also indicates that the addition of thermal stress may impair the resilience of these oysters to decreased pH. Overall, this study reveals the intracellular mechanisms that might enable marine calcifiers to endure the emergent, adverse seawater conditions resulting from climate change.

**Electronic supplementary material:**

The online version of this article (doi:10.1186/s12864-017-3818-z) contains supplementary material, which is available to authorized users.

## Background

Environmental change in marine ecosystems resulting from global climate change represents one of the most serious threats to marine organisms. Oceans are becoming warmer and more acidic as atmospheric concentrations of CO_2_ increase [1]. Marine organisms will need to acclimate or adapt in the face of these shifts in seawater chemistry and temperature. Many studies have described the negative impacts of ocean acidification (OA) and rising temperatures on the calcification, energy metabolism, reproduction, development and growth of marine calcifiers, such as oysters [2–8]. For instance, under elevated CO_2_ conditions, oysters exhibit weaker shells [9], higher mortality rates and lower soft tissue growth [10], as well as impaired immune functions (e.g. increase in apoptosis and reactive oxygen species production) [11]. Such pervasive impacts may ultimately affect the survival and sustainability of entire populations, especially when coupled with a warmer ocean.

The impacts of OA and rising temperatures on oysters also extend to the intracellular level. Proteomic studies have shown that CO_2_-driven OA (pH 7.3 to 7.87; 856 to 3000 μatm *p*CO_2_; 2- to 4-week exposure) alters the concentrations of proteins involved in antioxidant defence, stress responses, energy metabolism and the cytoskeleton in the oysters *Crassostrea gigas* (gills, hepatopancreas and larvae) [12–15], *Crassostrea virginica* (mantle) [16], *Crassostrea hongkongensis* (larvae) [17, 18] and *Saccostrea glomerata* (gills and hemocytes) [19, 20]. Similarly, transcriptional analysis of *C. virginica* oysters exposed to elevated CO_2_ (gill, mantle and hemocytes; pH 7.5 to 7.6; 2000 to 3500 μatm *p*CO_2_; 2-week exposure) has identified changes in the expression of genes associated with calcification, stress and immune responses [21, 22]. Increasing temperature also causes substantial changes in the molecular processes of marine organisms [23]. Heat shock proteins (Hsp) are one of the major protein families involved in the physiological response to thermal stress. Exposure to acute heat stress (+15 °C to 19 °C increases relative to current ambient conditions) has been shown to substantially increase (up to 2000-fold) Hsp expression in the gills of the Pacific oyster *C. gigas* (up to 12 h exposure) [24, 25]. Acute heat stress (+12 °C relative to ambient) also induced changes in the transcriptional levels of other genes involved in cellular homeostasis and protein synthesis (gills and mantle; 3–24 day exposure) [26]. At the protein level, increased concentrations of Hsp, as well as changes in the concentrations of proteins involved in energy metabolism, calcium binding and immune responses, were observed in the gills of *C. gigas* following exposure to temperature extremes (+20 °C relative to ambient; 1 h exposure) [27]. Although relevant given the broad temperature ranges oysters encounter in their natural environment, these studies have focused on acute heat stress (+1﻿2 °C to 20 °C relative to control) and so do not reflect near-future climate change scenarios where projected temperature increases span between 1.5 °C and 3.2 °C by the end of this century [1].

The transcriptomic response of oysters to a combination of low pH and increasing temperature has been assessed using next-generation sequencing (NGS) [28, 29]. The authors found that OA and the combination of both OA and increasing temperature have distinct effects on the intracellular systems of *C. virginica* and *C. gigas*. OA enhanced the expression of genes involved in antioxidant and metabolic processes in the gills and hepatopancreas of *C. virginica*. Both OA (up to −0.5 pH units relative to control) and increasing temperature (up to +3 °C relative to control) affected protein synthesis and processes involved in cell growth, as well as altering the expression of genes related to metabolism [29]. In the Pacific oyster, *C. gigas*, low pH (−0.4 pH units relative to control) enhanced the expression of immune response genes and the production of antioxidants, while the combination of both pH and thermal stresses (+5 °C relative to control) induced the expression of protease inhibitors and cytoskeleton-related genes in mantle tissue [28].

Recent evidence also suggests that oysters may be able to rapidly acclimate or adapt to such stressful conditions [6, 30, 31]. Molluscs from naturally CO_2_-enriched environments do not show the physiological effects typically induced when they are experimentally exposed to elevated CO_2_ [6, 31]. Moreover, environmental stressors associated with climate change often elicit different phenotypic responses in distinct populations within a species. Populations of *C. gigas* selectively bred for tolerance to acute heat stress (higher survival rates during experimentally-induced summer mortality events) show distinctive transcriptional profiles relative to populations that are sensitive to rising temperatures [32]. These heat-tolerant oysters exhibited lower expression levels of Hsp27, collagen, peroxinectin and S-crystallin, and higher expression of cystatin B following heat shock (+ 25 °C relative to ambient; 1–24 h exposure; gill tissue).

Elevated CO_2_ concentrations have also been shown to induce differential physiological and intracellular responses in a genetically-distinct population of Sydney rock oysters (*S. glomerata*). The B2 breeding line represents a unique genetic resource because of its capacity to cope with the effects of CO_2_ stress at the physiological level [30, 33]. This breeding line has been produced through mass selection for faster growth and resistance to the most significant oyster diseases in Australia [34]. Surprisingly, artificial selection for these characteristics appears to have coincidentally resulted in resilience against CO_2_-driven OA [30, 33]. Larvae from the B2 breeding line have higher survival rates and grow faster than wild type (non-selected) larvae following exposure to elevated CO_2_ (pH 7.90, 856 μatm *p*CO_2_) [30, 33]. B2 adults have higher standard metabolic rates (SMR) than wild type oysters under ambient conditions, and their SMR is further increased by CO_2_ stress [30].

Differential responses between populations of Sydney rock oysters are also evident at the molecular level. Distinctive changes in protein and gene regulation were found in B2 oysters after exposure to elevated CO_2_ (625 to 856 μatm *p*CO_2_, resulting in pH 7.78 to 7.84). Molecules involved in metabolism, oxidative stress, transcription, protein synthesis and modification, as well as signal transduction were the most affected by CO_2_-driven OA (gills and juvenile whole-body tissue) [19, 35]. The transgenerational responses of the B2 breeding line to CO_2_ stress have also been investigated at the level of gene expression. Exposure of B2 oysters to elevated CO_2_ over three consecutive generations (F2 juveniles) resulted in the up-regulation of genes associated with antioxidant defence, metabolism and the cytoskeleton (whole-body tissue) [35]. These findings suggest that the distinctive performance of B2 oysters might result from their capacity to regulate metabolic activity and control oxidative stress in face of elevated CO_2_ through differential regulation of molecules involved in these processes.

Despite the comprehensive characterisation of the responses of B2 oysters to near-future projected ocean pH, particularly at the physiological level, little is known about how these CO_2_-resilient oysters will respond to more realistic future scenarios that combine OA with increasing temperature. The current study addressed this deficit by describing the molecular processes of B2 oysters that are affected by the combination of OA and increasing temperature at both the protein and transcriptional levels. In the current study, we investigated the interactive effects of OA and increasing temperature on the molecular response of B2 oysters using a combination of proteomics and transcriptional analysis. This work is the first to explore the heritable potential of oysters for acclimation or adaptation to both climate change variables at the molecular level. This understanding will be crucial for assessing the performance of marine calcifiers and the sustainability of their populations in a changing ocean. It will also aid the development of marker-assisted breeding programs designed to safeguard oyster aquaculture industries as climate change progresses.

## Methods

### Oysters and exposure to elevated CO_2_ and temperature

Adult Sydney rock oysters (*Saccostrea glomerata*; Gould 1850) from the B2 breeding line were kindly provided by the Port Stephens Fisheries Institute (PSFI, Taylors Beach, NSW, Australia) of the New South Wales (NSW) Department of Primary Industries (DPI). The B2 breeding line has been bred for faster growth and resistance to the most significant oyster diseases in Australia: QX disease (causative agent: *Marteilia sydneyi*) and winter mortality syndrome (causative agent is currently under review [36]) [34]. Progenitors of the B2 line were established from parents collected from four rivers in NSW that had been affected by severe annual outbreaks of QX disease [37]. The breeding line has been produced by mass selection where fast growing survivors of disease epizootics are selected as parents for subsequent generations. The oysters used in this study were from the sixth generation of the B2 line. Surprisingly, this artificial selection for fast growth and disease resistance in the B2 line appears to have coincidentally conferred resilience against CO_2_-driven OA, such that B2 oysters can better withstand the impacts of elevated CO_2_ at the physiological level, compared to non-selected *S. glomerata* [30, 33].

Oysters (6.63 ± 0.71 cm shell length) were transferred to the Sydney Institute of Marine Science (Chowder Bay, NSW) and acclimated to aquarium conditions in a flow-through seawater system (0.5 L per minute, seawater filtered at 20 μm; corresponding to ambient conditions in Table 1). The nutritional supply from the flow-through seawater was supplemented every 3 days with a concentrated blend of microalgae (Shellfish Diet® 1800, Reed Mariculture Inc., 4.3 × 10^8^ algal cells per oyster). Following a 10-day acclimation, B2 oysters were exposed for 1 month to combinations of ambient (495 μatm *p*CO_2_, pH 8.09) or near-future elevated (1276 μatm *p*CO_2_, pH 7.72) CO_2_ concentrations with ambient (22.11 °C) or near-future elevated (25.38 °C) temperatures (Table 1). The elevated CO_2_ (mean decrease of 0.37 pH units) and temperature (mean increase of 3.26 °C) conditions are based on projected ocean surface increases by the year 2100 [1]. Seawater CO_2_ concentrations and temperatures were controlled by a custom-made system that included pneumatic components from Parker Hannifin (Castle Hill, NSW), CO_2_ sensors from Vaisala (Hawthorn, VIC) and a control system from Greenstar Building Automation & Citywide Electrical Services (Marrickville, NSW). CO_2_-enriched air and heated water were added into two header tanks, which supplied the treatments with reduced pH (elevated CO_2_). Each treatment comprised three replicate tanks (45 L), each of which contained 7 oysters (*n* = 21 oysters per treatment). Temperature, salinity and carbonate parameters (pH and total alkalinity) of seawater were monitored throughout the experiment (Table 1).

**Table 1:** Seawater chemistry during ocean acidification and warming trial

Parameter	Treatments			
	aCO_2_ + aT	aCO_2_ + eT	eCO_2_ + aT	eCO_2_ + eT
pH	8.10 ± 0.02	8.08 ± 0.02	7.70 ± 0.06	7.74 ± 0.08
Temperature (°C)	22.23 ± 0.16	25.86 ± 0.17	21.99 ± 0.30	24.90 ± 0.57
Salinity (ppt)	33.84 ± 0.02	33.84 ± 0.13	33.83 ± 0.45	33.83 ± 0.52
Total alkalinity (mmol kg^−1^ SW)	2.20 ± 0.02	2.20 ± 0.03	2.15 ± 0.03	2.14 ± 0.04
*p*CO_2_ (μatm)	473.5 ± 20.6	516.6 ± 22.3	1329.8 ± 181.2	1222.5 ± 210.9

Salinity, temperature and pH (NBS scale) were determined using a YSI 63 probe. Total alkalinity was measured in an automatic titrator (Metrohm 888 Titrando) and *p*CO_2_ was calculated using co2sys software [33]. Data are presented as mean ± SD (*n* = 66 per treatment; 3 tanks × 22 days). *Abbreviations*: *a* ambient, *e* elevated, *T* temperature

The experimental exposures produced a total of four treatments: aCO_2_ + aT (ambient CO_2_ and ambient temperature), aCO_2_ + eT (ambient CO_2_ and elevated temperature), eCO_2_ + aT (elevated CO_2_ and ambient temperature), and eCO_2_ + eT (elevated CO_2_ and elevated temperature). Following the one-month exposure to CO_2_ and/or thermal stress, oyster gills were collected and used for both proteomics and transcriptomics. Gills are amongst the tissues most immediately affected by changes in seawater quality, because they are metabolically active and represent the key interface between the organism and its environment. In addition, gill tissue is a tractable source of high quality RNA and protein in oysters. Hence, they have been used for proteomic and transcriptomic analyses in numerous previous studies (e.g. [12, 21, 27, 29]). For proteomics, oyster gills were immediately frozen at −80 °C (*n* = 15 per treatment), while gill samples for transcriptional analysis were stored in RNA later (Ambion) at −20 °C (*n* = 21 per treatment; including same oysters used for proteomics).

### RNA extraction and cDNA synthesis

Total RNA was extracted from approximately 100 mg of gill tissue (*n* = 21 oysters per treatment; individually sampled and processed) using TRI Reagent (Sigma-Aldrich), according to the manufacturer’s protocol. RNA was treated with DNase I (Promega) and further precipitated with 0.3 M sodium acetate (pH 5.5) and isopropanol. Concentration and quality of total RNA were checked with a Nanodrop spectrophotometer (Thermo Scientific NanoDrop 2000). Complementary DNA (cDNA) was synthesized from 1 μg of total RNA using ImProm-II™ Reverse Transcription System (Promega) and 0.5 μg of oligo(dT)_15_ in a 20 μl reaction volume.

### qPCR analysis

The transcriptional responses of B2 oysters to ocean acidification and warming were investigated by assessing the expression profiles of 60 genes involved in multiple biological processes. These genes were selected based on a previous study that explored the transcriptome of B2 oysters exposed to elevated CO_2_ (Goncalves et al., in preparation). Primers for quantitative (q) PCR analysis were designed to amplify the contigs most affected by ocean acidification (highest up- or down-regulation) (Additional file 1: Table S1).

Three microliter qPCR reactions were prepared in duplicate in 384-well plates using an epMotion® 5075 pipetting robot (Eppendorf) and an Echo® 550 Liquid Handler (Labcyte). Each reaction contained 1.5 μl KAPA SYBR® FAST qPCR Master Mix (Kapa Biosystems), 300 nM each primer, 0.3 μl PCR grade water and 1 μl cDNA template (diluted 1:9). qPCR assays were carried out on a LightCycler® 480 II (Roche). The cycling program used consisted of 3 min at 95 °C followed by 45 cycles of 95 °C for 10 s, 60 °C for 20 s and 72 °C for 6 s. At the end of the qPCR cycles, melting curve analysis was performed by collecting fluorescence data between 65 and 95 °C at 0.5 °C increments. Reaction efficiencies were calculated from standard curves generated in triplicate for each primer pair using five four-fold serial dilutions of a pool of cDNA samples (Additional file 1: Table S1). Cq values were obtained using the LightCycler® 480 Real-Time PCR System (version 1.5.1.62).

Gene expression stability of three potential reference genes was evaluated using the web-based RefFinder platform, which integrates results from different software tools [38]. Elongation factor 1 alpha (EF1α), β-actin and GAPDH were previously found to be stable under CO_2_ stress [35] and thus were tested in the current study. The geometric mean of these three genes combined were found to be the most stable combination (compared to each gene used individually or in pairs) and so all three genes were used as references (geNorm stability value = 0.102; NormFinder stability value = 0.051; Stability value by ΔCt method / Average SD = 0.17; BestKeeper stability value = 0.251). qPCR data are presented as changes in relative expression normalised with the geometric mean of the Cq values of EF1α, β-actin and GAPDH [39].

### Protein extraction

Proteins were isolated from approximately 100 mg of gill tissue (*n* = 15 per treatment; individually sampled and processed) using 1 ml of TRI Reagent (Sigma-Aldrich), according to the manufacturer’s protocol. After removal of RNA and DNA fractions, proteins were precipitated with acetone (3× volume) for 10 min followed by centrifugation for 10 min at 12000×g (4 °C). The resulting protein pellet was purified by multiple washes, following the protocol previously described by [19], with slight modifications. Briefly, protein pellets were washed by adding 1 ml of 0.3 M guanidine hydrochloride in 95% ethanol (3 × 10 min, room temperature) before centrifugation at 8000×g (4 °C). A final wash was carried out with 95% ethanol for 10 min followed by centrifugation as above. Protein pellets were air dried at room temperature and resuspended in 50 μl of rehydration buffer (7 M urea, 2 M thiourea, 4% CHAPS, 50 mM DTT and bromophenol blue).

Once purified, protein samples were quantified using Bradford reagent (Sigma-Aldrich) with BSA as the standard [40]. Equivalent amounts of proteins (30 μg) were pooled from five oysters taken from the same exposure tank, resulting in three biological replicates per treatment (3 pools of 5 oysters eac﻿h per treatment) and twelve 2D–gels across the four treatments (150 μg protein per gel).

### 2D gel electrophoresis

The proteomic responses of B2 oysters to ocean acidification and warming were assessed by two-dimensional gel electrophoresis (2DE). A total of three gels were run per treatment (representing each of the three replicate tanks per treatment). Proteins (150 μg in 125 μl of rehydration buffer containing 0.2% pharmalytes) were immobilised in pH linear gradient gel strips (7 cm, pH 4–7; ReadyStrip™ IPG Strips, Bio-Rad) by overnight passive rehydration. Isoelectric focusing (IEF) was performed using an IPGphor IEF System (GE Healthcare) at 100 V for 2 h, 250 V for 20 min, a gradient up to 5000 V for 2 h and then 5000 V for 2 h. Following IEF, gel strips were equilibrated for 20 min in equilibration buffer I (1% DTT, 75 mM of 1.5 M Tris-HCl pH 8.8, 6 M urea, 30% glycerol, 2% SDS, bromophenol blue) and then for 20 min in equilibration buffer II (2.5% iodoacetamide instead of 1% DTT in equilibration buffer I). Second dimension separation was conducted using 12% Mini-PROTEAN® TGX™ Precast Protein Gels (Bio-Rad) in a Mini-PROTEAN® Tetra Vertical Electrophoresis System (Bio-Rad). After electrophoresis, gels were stained with blue silver [41] and visualised using a ChemiDoc XRS+ (Bio-Rad). Quantitative image analysis of protein spots was performed by PDQuest 2-D Analysis Software (Bio-Rad).

### In-gel digestion of differentially regulated proteins

The protein spots found to be differentially regulated by elevated CO_2_ and/or elevated temperature were excised manually and subjected to in-gel digestion. A fresh set of 2D gels was used for spot picking. Gel pieces were washed with 100 mM ammonium bicarbonate and then destained with 50% acetonitrile in 50 mM ammonium bicarbonate (multiple washes for 10 min each). Following destaining, gel pieces were dehydrated in 100% acetonitrile for 5 min before being air dried. They were then reduced with 10 mM DTT in 100 mM ammonium bicarbonate at 56 °C for 1 h, alkylated with 55 mM iodoacetamide in 100 mM ammonium bicarbonate (45 min at room temperature) and further washed and dehydrated as above. Trypsin (12.5 ng/μl in 50 mM ammonium bicarbonate; Promega) was added to each gel piece and the mixture was incubated first for 30 min at 4 °C and then overnight at 37 °C. After overnight digestion, gel pieces were washed twice in 50% acetonitrile and 2% formic acid for 30 min to recover the peptide-containing supernatant (50 to 60 μl final volume). Supernatants were concentrated to 12 μl in a vacuum concentrator (Concentrator Plus, Eppendorf) and then centrifuged at 12000×g for 10 min to remove microparticles.

### Protein characterisation by mass spectrometry

Trypsin-digested peptides were analysed by nanoflow liquid chromatography-tandem mass spectrometry (LC-MS/MS) using a Finnigan Surveyor MS Pump Plus coupled to a Finnigan LTQ XL™ Linear Ion Trap Mass Spectrometer (Thermo Scientific). Chromatography was performed in reversed-phase peptide trap columns packed to approximately 9 cm (100 μm ID) with Magic C18AQ (5 μm, 200 Å, Michrom Bioresources), in a fused silica capillary with an integrated electrospray tip, coupled to pre-columns packed with PS-DVB resin (3 cm, 100 μm ID, Agilent Technologies). A 1.8 kV electrospray voltage was applied through a liquid junction up-stream of the C18 column. Sample injection was carried out using an EASY-nLC II (Thermo Scientific). Peptides were washed with buffer A (2% acetonitrile, 0.1% formic acid) for 2 min at 140 nl/min and then eluted from the column with 0–20% gradient of buffer B (95% acetonitrile, 0.1% formic acid) at 140 nl/min for 38 min. Following peptide elution, the column was washed with 95% buffer B at 140 nl/min for 10 min. Spectra were acquired for 60 min in positive ion mode for the scan range of 400 m/z to 1500 m/z. Automated peak recognition, dynamic exclusion and MS/MS of the top nine most intense precursor ions at 35% normalisation collision energy were performed using Xcalibur™ software (version 2.06, Thermo Scientific).

Mass spectrometry data files were analysed using Global Proteome Machine (GPM) software (version 2012.05.01) and the X!Tandem algorithm [42, 43]. Files were searched against a non-redundant (nr) protein database for Mollusca (downloaded from NCBI in March 2015), using default settings. The Mollusca nr database contained 378,914 sequences, including those derived from the *C. gigas* genome, in addition to common human and trypsin peptide contaminants. Spectra were also searched against a reversed sequence database for estimation of false discovery rates (FDR). Peptides that yielded log(e) values ≤ −10 and at least five spectral counts were retained for protein characterisation.

### Statistical analyses

The statistical significance of differences between treatments for proteomic and transcriptional data was assessed by a linear model, considering the ambient control condition as the reference (*p* < 0.05), and by the significance analysis of microarrays (SAM; FDR < 10%). Statistical analysis on proteomic data was performed on the normalised intensities of protein spots from 2D gels. Relative gene expression data used for statistical analysis were calculated by normalising Cq values of each target gene with the geometric mean of the Cq values of EF1α, β-actin and GAPDH (reference genes) [39]. Tests were carried out using the mean response of oysters from the same exposure tank, such that each of the 3 tanks per treatment was considered to be an independent biological replicate. Three tanks per treatment each containing a pool of 5 oysters were used for proteomic analysis, while 3 tanks per treatment each containing 7 oysters (individual samples) were used for gene expression analysis.

Fold differences for each differentially expressed protein or gene were determined by comparing the mean normalised protein spot intensities or mean relative expression of each treatment where at least one parameter (CO_2_ or temperature) was elevated relative to those of ambient control conditions (aCO_2_ + aT). Fold change (FC) values >1 reflect up-regulation in the elevated treatment relative to ambient controls. FC values <1 reflect down-regulation in the elevated treatment. Differences between CO_2_ and temperature conditions were also visualised in non-metric multi-dimensional scaling (NMDS) plots. NMDS was based on Bray-Curtis similarity coefficients calculated from normalised intensities of differentially regulated proteins or differentially expressed genes (absolute fold-change ≥1.5).

Differences in seawater parameters (temperature, pH, *p*CO_2_, salinity and total alkalinity) between the elevated treatments were assessed by Student’s t test, comparing the conditions in tanks sharing the same elevated parameter (e.g. elevated CO_2_ + ambient temperature vs. elevated CO_2_ + elevated temperature treatment; and ambient CO_2_ + elevated temperature vs. elevated CO_2_ + elevated temperature).

## Results

### Experimental conditions for exposure to OA and warming

Seawater chemical and physical parameters were assessed throughout the experimental exposures (Table 1). Tanks for elevated CO_2_ and for elevated temperature treatments were supplied by independent seawater reservoirs, which resulted in differences in pH and temperature among treatments. Such variation in pH and temperature was also reflected by small differences in seawater carbonate chemistry, since *p*CO_2_ concentration was calculated based upon measurements of these two variables (in addition to salinity and total alkalinity, which did not show significant variability). Seawater pH was 0.04 units lower in elevated CO_2_ + ambient temperature (eCO_2_ + aT) relative to the elevated CO_2_ + elevated temperature treatment (eCO_2_ + eT) (*p* = 0.04, two-tailed t-test). As a result, *p*CO_2_ concentration was higher (+107 μatm) in elevated CO_2_ + ambient temperature (eCO_2_ + aT) tanks relative to the elevated CO_2_ + elevated temperature condition (eCO_2_ + eT) (Table 1). However, this difference in *p*CO_2_ concentrations between the elevated CO_2_ treatments was not statistically significant (*p* = 0.08, two-tailed t-test).

Similar variation was also evident between the elevated temperature treatments. Tanks for ambient CO_2_ + elevated temperature (aCO_2_ + eT) and elevated CO_2_ + elevated temperature (eCO_2_ + eT) were also supplied by independent flow-through seawater systems, which led to differences in temperature between treatments. Tanks for ambient CO_2_ + elevated temperature (aCO_2_ + eT) treatment were 0.96 °C warmer than those for elevated CO_2_ + elevated temperature (eCO_2_ + eT) (*p* = 3.88e-11, two-tailed t-test) (Table 1).

### Molecular profiles of oysters in response to ocean acidification and warming

Ocean acidification (OA) and warming induced substantial changes in the proteomes and gene expression of B2 oysters. The differential proteomes of oysters in response to elevated CO_2_ and/or elevated temperature were resolved by 2D gel electrophoresis. An average of 345 protein spots was detected across the 2D gels, with molecular weights (MW) ranging from 12 to 200 kDa and isoelectric points (pI) ranging from 4 to 7.

**Fig. 1:**
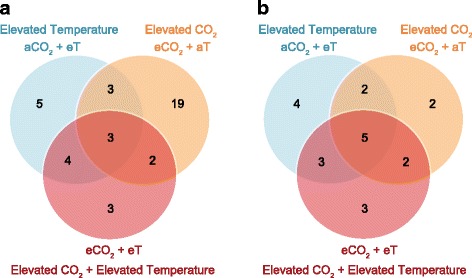
Effects of ocean acidification and warming on the molecular responses of oysters. Venn diagrams showing the number of proteins (**a**) and genes (**b**) that were differentially regulated in oyster gills in response to elevated CO_2_ and/or elevated temperature. Differential regulation was determined by comparing spot normalised intensities or relative gene expression between each treatment and the control, ambient condition. aCO_2_ + eT: ambient CO_2_ and elevated temperature; eCO_2_ + aT: elevated CO_2_ and ambient temperature; and eCO_2_ + eT: elevated CO_2_ and elevated temperature

**Fig. 2:**
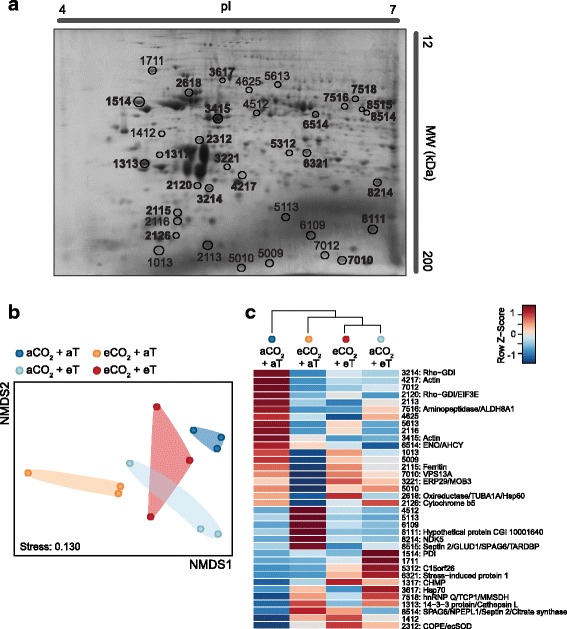
Proteomic responses of oysters to ocean acidification and warming. **a** A representative gill proteome map of B2 line oysters. Protein spots differentially regulated due to pH and/or temperature treatments are highlighted. Numbers associated with each protein spot correspond to arbitrary identifiers generated by PDQuest during image analysis. Numbers in bold indicate the 23 protein spots that were successfully identified by mass spectrometry. pI, isoelectric point; MW, molecular weight in kDa. **b** Non-metric multidimensional scaling (NMDS) plot summarising the cumulative normalised intensities of 23 differentially regulated protein spots among B2 oysters exposed to elevated CO_2_ and/or elevated temperature (T). Each point represents the spot intensity levels of pooled samples containing 5 oysters each per condition. **c** Heat map of mean normalised intensities of differentially regulated proteins. Proteins are identified by their spot numbers followed by their putative identifications obtained using mass spectrometry. Protein names and fold differences are detailed in Additional file 2: Table S2. aCO_2_ + aT: ambient CO_2_ and ambient temperature; aCO_2_ + eT: ambient CO_2_ and elevated temperature; eCO_2_ + aT: elevated CO_2_ and ambient temperature; and eCO_2_ + eT: elevated CO_2_ and elevated temperature

Of the 345 protein spots identified, 39 were found to differ significantly in intensity (*p* < 0.05 or FDR < 10%) in at least one of the elevated CO_2_ and/or elevated temperature treatments, relative to control conditions (aCO_2_ + aT). Of these, three differential proteins were common to all three elevated treatments (aCO_2_ + eT, eCO_2_ + aT and eCO_2_ + eT), while 27 were unique to particular treatments. Of these, most (19; 70%) were unique to the elevated CO_2_ + ambient temperature treatment (eCO_2_ + aT) (Fig. 1a). Mass spectrometry was performed on 36 differentially regulated proteins (proteins spots are highlighted in Fig. 2a). Twenty-three proteins (64%) were successfully identified based on homology to a Mollusca non-redundant (nr) database (Additional file 2: Table S2).

The transcriptional responses of B2 oysters to elevated CO_2_ and/or elevated temperature were analysed by qPCR. We evaluated the relative expression profiles of 60 genes that have been previously shown to be differentially regulated following transgenerational exposure of B2 oysters to CO_2_ stress (Goncalves et al., in preparation). Twenty-one genes (35%) were found to be affected by one or both stresses (elevated CO_2_ and/or elevated temperature). Figure 1b shows the number of differentially expressed genes that were unique to particular treatments and those that were affected by one or more treatments. Five differentially regulated genes were common to all three elevated treatments (aCO_2_ + eT, eCO_2_ + aT and eCO_2_ + eT), while nine were unique to particular treatments.

### Effects of ocean acidification

**Fig. 3:**
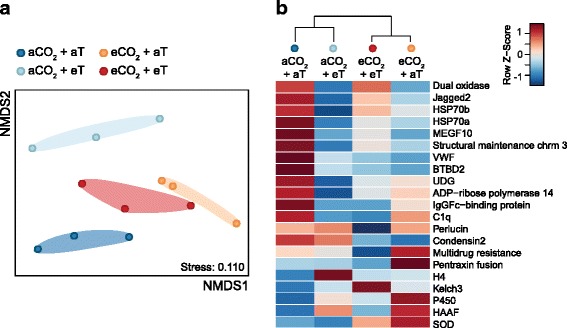
Transcriptional responses of oysters to ocean acidification and warming. **a** Non-metric multidimensional scaling (NMDS) plot showing the cumulative expression profiles of differentially regulated genes among B2 oysters (gill tissue) exposed to elevated CO_2_ and/or elevated temperature (T). Each point represents the mean relative expression levels of oysters from the same exposure tank (average of 7 oysters for each of the 3 exposure tanks per condition). **b** Heat map of mean relative expression of differentially regulated genes assessed by qPCR. Gene names and their associated cellular functions are detailed in Additional file 1: Table S1. aCO_2_ + aT: ambient CO_2_ and ambient temperature; aCO_2_ + eT: ambient CO_2_ and elevated temperature; eCO_2_ + aT: elevated CO_2_ and ambient temperature; and eCO_2_ + eT: elevated CO_2_ and elevated temperature

The exposure of oysters to elevated CO_2_ altered the expression of numerous individual proteins and genes. This is reflected by the clear spatial discrimination of cumulative protein and gene expression profiles between CO_2_-exposed oysters (eCO_2_ + aT) and the other treatments (Fig. 2b and Fig. 3a). Elevated CO_2_ by itself (eCO_2_ + aT) significantly affected the expression of 27 proteins and 11 transcripts (relative to aCO_2_ + aT). (Fig. 1). This represented the highest number of differentially regulated proteins among all of the CO_2_ and temperature treatments. NMDS plot incorporating data for differential proteins reveals that the eCO_2_ + aT treatment had the most substantial response relative to ambient controls (aCO_2_ + aT) (Fig. 2b). The majority of differentially regulated proteins (63%) and genes (64%) were less abundant in eCO_2_ + aT oysters relative to the control treatment (aCO_2_ + aT). In this comparison, the proteins found in lower abundance included actin (0.23-fold), ferritin (0.33-fold), cytochrome b5 (0.40-fold), vacuolar protein sorting-associated protein 13A (VPS13A; 0.49-fold) and Rho GDP dissociation inhibitor 1 (Rho-GDI; 0.50-fold) (Fig. 2c). Similarly, genes encoding von Willebrand factor D (VWF) and EGF domain-containing protein (0.17-fold), structural maintenance of chromosomes protein 3 (SMC3; 0.27-fold), multiple epidermal growth factor-like domains protein 10 (MEGF10; 0.36-fold), dual oxidase (0.54-fold) and heat shock 70 kDa protein 12a (Hsp70a; 0.57-fold) were down-regulated by CO_2_ stress (Fig. 3b).

Few proteins and genes were up-regulated by elevated CO_2_ alone (eCO_2_ + aT). Heterogeneous nuclear ribonucleoprotein Q (hnRNP Q; 2.66-fold), nucleoside diphosphate kinase-like protein 5 (NDK5; 2.51-fold), 14–3-3 protein (2.26-fold), septin 2 (2.15-fold) and sperm associated antigen 6 (SPAG6; 2.00-fold) were found at higher concentrations in eCO_2_ + aT oysters when compared to the aCO_2_ + aT treatment (Fig. 2c). At the transcriptional level, extracellular superoxide dismutase (ecSOD; 2.46-fold), cytochrome P450 CYP1A1 (6.88-fold), pentraxin fusion protein (2.44-fold) and hemagglutinin/amebocyte aggregation factor (HAAF; 1.83-fold) had higher expression levels in CO_2_-exposed oysters relative to controls (Fig. 3b).

### Effects of ocean warming

Thermal stress by itself (aCO_2_ + eT) also affected the gill proteomes and transcriptional responses of B2 oysters. Oysters exposed to elevated temperature alone (aCO_2_ + eT) exhibited protein and gene expression profiles that were clearly different from those of oysters held under ambient conditions (aCO_2_ + aT) and oysters exposed to elevated CO_2_ alone (eCO_2_ + aT) (Fig. 2b and Fig. 3a). The transcriptional response of aCO_2_ + eT oysters was also distinct from the combined eCO_2_ + eT treatment (Fig. 3a). However, this distinction was less apparent in their proteomic responses. NMDS of differentially regulated proteins showed substantial overlap between the aCO_2_ + eT and eCO_2_ + eT treatments (Fig. 2b).

Elevated temperature alone (aCO_2_ + eT) altered the regulation of 15 proteins and 14 genes, affecting a greater number of genes compared to elevated CO_2_ alone (Fig. 1). Most of the differentially regulated proteins (60%) and genes (79%) were found at lower concentrations in oysters exposed to elevated temperature (aCO_2_ + eT) relative to control conditions (aCO_2_ + aT). In this comparison, down-regulated proteins included enolase (0.42-fold), Rho-GDI (0.49-fold) and actin (0.51-fold) (Fig. 2c), while uracil-DNA glycosylase (UDG; 0.42-fold), dual oxidase (0.44-fold) and Hsp70 (0.42-fold for Hsp70a; 0.49-fold for Hsp70b) were among the down-regulated transcripts (Fig. 3b). Conversely, protein disulphide isomerase (PDI; 3.85-fold) and stress-induced protein 1 (2.50-fold) were found in higher concentrations following heat stress (Fig. 2c), as well as the transcripts encoding CYP1A1 (3.93-fold), histone H4 (2.20-fold) and HAAF (1.64-fold) (Fig. 3b).

### Combined effects of ocean acidification and warming

Oysters exposed to both elevated CO_2_ and elevated temperature (eCO_2_ + eT) exhibited a unique gene expression profile that was different from all other treatments (Fig. 3a). Conversely, the differential proteomes of oysters exposed to both CO_2_ and heat stresses (eCO_2_ + eT) overlapped with the proteomes of oysters exposed to heat stress only (aCO_2_ + eT) (Fig. 2b).

The combination of elevated CO_2_ and elevated temperature changed the expression patterns of 12 proteins and 13 genes (Fig. 1). The majority of differentially regulated proteins (58%) and genes (77%) were found at lower concentrations in eCO_2_ + eT relative to ambient conditions (aCO_2_ + aT). Proteins found in lower abundance in eCO_2_ + eT oysters included actin (0.41-fold), Rho-GDI (0.67-fold) and protein spot 7516 (0.47-fold) (Fig. 2c). Genes down-regulated by elevated CO_2_ and elevated temperature included VWF (0.26-fold), perlucin (0.60-fold), multidrug resistance protein 1 (0.63-fold) and IgGFc-binding protein (0.63-fold) (Fig. 3b). In contrast, charged multivesicular body protein 4c (2.02-fold), stress-induced protein 1 (1.88-fold) and protein spot 2312 (2.85-fold) were found at higher concentrations in eCO_2_ + eT oysters (Fig. 2c). At the transcriptional level, ecSOD (1.90-fold), CYP1A1 (3.28-fold) and kelch 3 (1.55-fold) showed higher expression levels in eCO_2_ + eT oysters relative to controls (Fig. 3b).

Only 3 proteins and 5 genes were affected by all stress treatments (elevated CO_2_ alone, elevated temperature alone, and the combination of elevated CO_2_ and elevated temperature) (Fig. 1). Rho-GDI, actin and an unidentified protein (spot no. 2311) were found in lower concentrations in oysters exposed to all three treatments (0.22 to 0.67-fold) relative to oysters held under ambient conditions (Fig. 2c). At the transcriptional level, MEG10, Hsp70a, SMC3 and VWF were down-regulated by all of the treatments (0.01 to 0.65-fold), while CYP1A1 transcripts were more abundant under CO_2_ and/or thermal stresses (3.28 to 6.88-fold) (Fig. 3b).

## Discussion

B2 oysters represent a valuable genetic resource because of their capacity to withstand the impacts of CO_2_ stress at the physiological level [30, 33]. They also appear to be capable of rapid acclimation or adaptation, responding faster than wild type oysters in the face of ocean acidification (OA) [35]. These features make this population a potential tool for climate-proofing Sydney rock oyster aquaculture and restoring natural oyster beds. The current study characterised the molecular profile of B2 oysters responding to realistic near-future scenarios that combine OA with ocean warming. By assessing the intracellular effects of elevated CO_2_ and increasing temperature, we found substantial changes in protein concentrations and gene expression in oyster gills following exposure to one or both stressors. Although the combination of these two stressors also altered the regulatory patterns of proteins and genes, the concurrent effects of elevated CO_2_ and temperature were not shown to be synergistic or additive.

### Overlapping responses to OA, increasing temperature and the combined stressors

A suite of proteins and genes showed substantial changes in expression in response to all stress treatments - elevated CO_2_ alone, elevated temperature alone, and combination of elevated CO_2_ and elevated temperature. This overlap in stress responses among treatments suggests that a similar set of molecular processes is recruited in the face of OA and warming in B2 oysters. The intracellular processes simultaneously affected by OA and/or increasing temperature were the control of redox balance, maintenance of cellular homeostasis, stress responses and the cytoskeleton. Differentially regulated molecules included Rho GDP dissociation inhibitor 1 (Rho-GDI), actin, Hsp70a, von Willebrand factor (VWF) D and EGF domain-containing protein, multiple epidermal growth factor-like domains proteins 10 (MEG10), structural maintenance of chromosomes 3 (SMC3) and a cytochrome P450 encoded by CYP1A1.

Rho-GDI and actin are of particular interest in t﻿he con﻿text of clima﻿te change resilience. Both proteins were found in lower concentrations in stress-exposed oysters relative to control conditions. Rho-GDI is an inhibitor of the small GTPase Rho, and is involved in oxidative stress and cytoskeleton organisation. Following interaction of Rho-GDI with membrane proteins, this complex activates NADPH oxidase, which in turn initiates the production of reactive oxygen species (ROS) [44]. Lower abundance of Rho-GDI has been observed in the mussel *Mytilus galloprovincialis* recovering from hyposaline stress [45]. The down-regulation of Rho-GDI found in our study may be associated with the control of oxidative damage that is typically induced by OA and rising temperature in oysters. The low abundance of Rho-GDI could prevent the formation of ROS in response to CO_2_ and/or thermal stresses in B2 oysters by reducing the activation of NADPH oxidase. Alternatively, the down-regulation of this protein could increase the activity of Rho GTPase, leading to cytoskeletal modifications. In this context, it is relevant that the major cytoskeletal protein, actin, was also found at lower concentrations in oysters exposed to any the three stress treatments. Actins are highly abundant structural proteins that play a fundamental role in the division, shape and mobility of eukaryotic cells. The changes observed in actin abundance could be related to cytoskeletal remodelling due to intracellular stress [23, 46].

At the transcriptional level, a gene encoding the stress response protein Hsp70 was also down-regulated in oysters exposed to any of the three stress treatments (OA, increased temperature and the combination of these stresses). Heat shock proteins, including Hsp70, are among the most abundant intracellular proteins protecting cells from stress-induced damage. Hsp70s act as molecular chaperones, promoting protein folding, membrane translocation, degradation of misfolded proteins and maintenance of cellular homeostasis [47]. Down-regulation of Hsp70s following exposure to elevated CO_2_ has been previously shown in the oysters *C. virginica* and *S. glomerata* (B2 line) (pH 7.59 to 7.88; one-month exposure; hemocytes and gills) [19, 22]. In the pearl oyster, *Pinctada fucata*, Hsp70 mRNA levels increased immediately after exposure to elevated CO_2_ alone (pH 7.70, 1425 μatm *p*CO_2_), elevated temperature (+3 **°**C relative to control) alone and a combination of these stressors. However, Hsp70 expression in the mantle of *P. fucata* decreased after 4 days of stress exposure (relative to non-exposed oysters) [48]. In line with these studies, our findings suggest that longer exposures to stressful seawater conditions cause a decrease in the concentration of Hsp70. Such reductions in the expression of Hsp70, as well as of Rho-GDI and actin, may indicate that B2 oysters are prone to protein denaturation and cytoskeleton remodelling caused by CO_2_ and thermal stresses.

Exposure to OA, increased temperature and the combination of both stresses also altered expression of genes involved in the maintenance of cellular homeostasis and the cell cycle. Genes encoding proteins that contain von Willebrand factor D (VWF) and EGF-like domains were substantially down-regulated in exposed B2 oysters. Von Willebrand factor D (VWF) and EGF domain-containing proteins are involved in the maintenance of cellular homeostasis, as well as in protein-protein interactions and the assembly of protein subunits [49]. MEGF10, another gene down-regulated by all three stress treatments, encodes a membrane protein involved in phagocytosis of apoptotic cells, cell adhesion and motility. The expression of MEGF10 was down-regulated by hypoxia and hypo-osmotic stress in the Pacific oyster *C. gigas* (pool of tissues) [50]. A gene encoding SMC3 was also down-regulated by CO_2_ and/or thermal stresses in the current study. SMC3 is a central component responsible for maintaining chromosome cohesion during cell division. In addition to its involvement in DNA replication and the cell cycle, SMC3 is associated with DNA repair [51]. To our knowledge, this is the first report showing differential regulation of SMC3 in response to stress in oysters. Due to this lack of information about the role that SMC3 plays in oysters, it is difficult to explain the implication of its low expression in stressed oysters. In contrast, CYP1A1 was the only molecule that showed increased expression in response to all three stress treatments. CYP1A1 is a member of the cytochrome P450 superfamily and encodes a protein that is involved in a variety of detoxification and endogenous processes, being the major form induced by xenobiotics, including dioxins, polycyclic aromatic hydrocarbons (PAHs) and polychlorinated biphenyls (PCBs) [52, 53]. Induction of CYP1A1 transcription has been frequently reported after exposure to environmental stress, particularly chemical contaminants [53, 54]. The down-regulation of genes involved in the cell cycle and maintenance of cellular homeostasis, along with the increased expression of CYP1A1, indicate that changes in seawater chemical and/or physical characteristics could have substantial effects on oyster growth and development, and their ability to respond to stress.

### Molecular responses induced by OA

Despite the similarities in responses to all three stress treatments (OA alone, increased temperature alone and the combination of these two stressors), each of the treatments produced additional unique impacts on different proteins and genes. CO_2_ stress by itself induced the greatest changes at the protein level among all of the treatments, both in terms of number of proteins affected and magnitude of those changes. Previous studies have shown that molluscs are particularly susceptible to OA [4–6, 15]. Changes in seawater chemistry have negative effects on growth, development, survival, calcification and acid-base regulation in different species of molluscs [4, 5, 7]. These changes are likely to be a reflection of complex rearrangements at the molecular level that ultimately result in physiological impacts [55]. Increasing temperature, in contrast, has not been shown to produce such strong impacts on the performance of molluscs [7]. Among the genes/proteins affected by CO_2_ stress alone in the current study, a number are associated with responses to stress, redox balance and cytoskeleton organisation. In the context of the molecules involved in redox balance, cytochrome b5 and ferritin were found at lower concentrations following exposure to elevated CO_2_. Both proteins participate in reduction-oxidation reactions and iron homeostasis. Given that these processes are linked to energy metabolism (through expression of Krebs cycle enzymes, for example), lower concentrations of cytochrome b5 and ferritin may be associated with shifts in energy requirements induced by CO_2_ stress [56].

Other stress-related genes affected by elevated CO_2_ alone included extracellular superoxide dismutase (ecSOD) and septin 2 (both up-regulated). EcSOD participates in ROS scavenging while septin is required for normal organization of the actin cytoskeleton [16, 57]. Increased concentrations of ecSOD and septin 2 may be related to the capacity of B2 oysters to cope with oxidative damage typically induced by OA. They could mitigate damage to the actin cytoskeleton and other fundamental cellular components, minimising cell death through apoptosis. Overall, these findings suggest that B2 oysters may be able to withstand and limit the adverse impacts of CO_2_ stress on their physiological responses (i.e. growth, survival and metabolism) by hindering disturbance of the cellular redox balance and controlling cellular homeostasis.

### Molecular responses to increasing temperature

Thermal stress alone also induced unique, substantial changes in the regulation of proteins and genes in B2 oysters. While elevated CO_2_ was the stress that altered the largest number of proteins, elevated temperature was responsible for the greatest changes at the transcriptional level. Thermal stress reduced the abundance of enolase and uracil-DNA glycosylase transcripts, but increased the levels of protein disulphide isomerase and stress-induced protein 1. Enolase participates in carbohydrate metabolism, specifically glycolysis, and may also be involved in stress responses [58]. Uracil-DNA glycosylase (UDG) is a major component of the cellular machinery responsible for DNA repair and prevention of DNA damage [59, 60]. Very little information is available on the biological role and transcriptional regulation of enolase and UDG in molluscs responding to stress. However, our data might suggest that thermal stress reduces the ability of cells to control oxidative damage and resulting mutation, with consequences for the prevention of heat-induced cell death.

Protein disulphide isomerase (PDI) and stress-induced protein 1 were among the molecules whose expression was increased by thermal stress. PDI is a molecular chaperone found in the endoplasmic reticulum. It is involved in protein folding, assembly and post-translation modifications, including those induced by ROS [61]. Higher levels of PDI have also been observed in thermally-stressed sea cucumbers [62] and in larvae from Pacific oysters (*C. gigas*) exposed to the combination of elevated temperature (+2 **°**C relative to control) and elevated CO_2_ (pH 7.9, 656 μatm *p*CO_2_) [63]. In line with our results, stress-induced protein 1, another molecular chaperone, was also found to be up-regulated in the gills of *C. gigas* following acute heat stress (+ 20 **°**C for 1 h) [27]. It has been extensively reported that elevated temperature triggers oxidative stress in different species of marine invertebrates [23, 24, 26]. This response is often elicited by an imbalance between the production and neutralisation of ROS due to heat-induced metabolic shift and increased oxygen requirements. Our findings suggest that the increased expression of molecular chaperones may preserve correct conformation and stability of a number of proteins, in an attempt to prevent damage from thermally-driven stress.

### Molecular responses to the combination of OA and warming

The combination of OA and warming induced specific changes that were not evident when oysters were exposed to elevated CO_2_ or temperature alone. The combined stressors caused down-regulation of perlucin and multidrug resistance protein 1. Perlucin is a C-type lectin involved in nucleation of calcium carbonate ions during shell formation [64–66]. Although the expression of perlucin may be expected to be altered by CO_2_-driven OA, no changes in its mRNA levels were observed in the mantle of the mussel *Mytilus edulis* after exposure to elevated CO_2_ (pH 7.8, 766 μatm *p*CO_2_, 60-day exposure) [67, 68]. In contrast, higher levels of perlucin was observed in gills of B2 oysters following transgenerational exposure to CO_2_ stress alone (pH 7.77, 1067 μatm *p*CO_2_, 4-week exposure) (Goncalves et al., in preparation). Therefore, it appears that B2 oysters could be able to maintain carbonate homeostasis and shell deposition under CO_2_ stress alone, but their calcification process may be hampered when increased temperature is combined to CO_2_ stress. Multidrug resistance proteins play a role in detoxifying various toxic agents in marine organisms due to their ability to transport xenobiotic conjugates and metabolites [69]. Our findings indicate that multidrug resistance protein 1 may also participate in oyster responses to OA and warming.

Combined, the substantial changes in protein and gene expression observed in this study suggest that the inducible responses of oysters to near-future changes in seawater temperature and pH may depend upon energy reallocation. Such reallocation could regulate fundamental cellular processes, including control of oxidative damage, maintenance of cellular homeostasis and immune responses. Although testing the physiological outcomes of this stress-induced energy reallocation was beyond the scope of our study, it is likely that these intracellular changes have an impact on oyster growth and survival (as previously shown by [10, 30, 70]) as well as on the immune functions and response to infectious diseases. Intracellular changes induced by thermal stress in Pacific oysters (*C. gigas*) have been related to potential susceptibility to infectious diseases [71–73].

### Concurrent effects of OA and warming are not synergistic or additive

Numerous studies suggest that exposure to elevated CO_2_ enhances the responsiveness of marine organisms to thermal stress and vice versa [74–76]. However, our data did not reveal such a synergistic or additive interaction at the molecular level. In the context of the current study, synergistic or additive effects would have been reflected in more substantial responses, in terms of the number or fold changes of differentially expressed genes and proteins, when the combined effects of elevated CO_2_ and temperature were compared to those stressors in isolation. By this definition, our data did not identify synergistic or additive effects of elevated CO_2_ and temperature. This lack of additive or synergistic interactions may be due to limitations in our experimental design or to potentially antagonistic effects of OA and increasing temperature in B2 oysters. Variability in pH and temperatures (but not in *p*CO_2_ concentrations) were observed among the different treatments as a consequence of our experimental design. Tanks for elevated CO_2_ and elevated temperature treatments were supplied by independent seawater reservoirs. This resulted in unintended, albeit minor differences in seawater chemistry and temperature among the treatments. These differences may have prevented us from identifying synergistic or additive effects by CO_2_ and thermal stresses.

The lack of a synergistic or additive response might also have been due to the differential performance of B2 oysters under climate change-associated conditions. If that is the case, the combination of OA and increased temperature would induce antagonistic effects in these oysters. Previous studies have reported that thermal stress ameliorates some impacts of elevated CO_2_. Increasing temperature (+2 **°**C to 3.2 **°**C relative to control conditions) minimised the negative effects of elevated CO_2_ (resulting in pH 7.79 to 7.90) on growth and calcification rate of *C. gigas* larvae [63], and on calcification of coral reefs [77] and the sea star *Pisaster ochraceus* [78]. Similarly, a recent meta-analysis assessing the physiological responses of marine organisms to OA and warming revealed restorative effects of the combined stressors on the growth of echinoderms and calcifying phytoplankton, and on photosynthesis of calcifying and non-calcifying marine autotrophs. It has also been shown that OA alone negatively affects both calcification and photosynthesis in corals, while these processes were not disturbed by the combination of OA and increased temperature [7]. In line with these findings, our data suggest that the addition of warming may alleviate the effects of acidification on the molecular responses of oysters. Alternatively, our results might imply that the combination of elevated CO_2_ and temperature cumulatively overwhelms the stress response system of B2 oysters, so that their intracellular processes more closely resemble those of oysters held under ambient conditions compared to those exposed to elevated CO_2_ or temperature in isolation.

## Conclusions

The current study has investigated the impacts of ocean acidification and warming on gill proteomes and transcriptional responses of B2 oysters. Our findings show that low seawater pH and elevated temperature affect a variety of biological processes at the protein and transcriptional levels. These effects are not additive or synergistic and may be antagonistic, even though many of the same intracellular processes are affected by elevated CO_2_ and elevated temperature when the stressors are applied separately. The differential responses observed in oysters exposed to a combination of stressors indicate that the inherent capacity of B2 oysters to cope with elevated CO_2_ that was described at the physiological level may not be substantially relevant once thermal stress is added in marine environments. Further studies are required to clarify the complex biological outcomes of the concurrent exposure to ocean acidification and warming, as well as to elucidate the potential of B2 oysters to further acclimate or adapt to such stressful conditions.

## Additional files

Additional file 1: Table S1. Primers used for qPCR analysis. (XLSX 15 kb)

Additional file 2: Table S2. List of proteins identified by mass spectrometry that were differentially regulated by CO_2_ and/or thermal stress. Fold changes (FC) were calculated from the mean normalised intensities of protein spots relative to the control, ambient condition (aCO_2_ + aT). FC values >1 reflect up-regulation in the elevated treatment relative to ambient controls. FC values <1 reflect down-regulation in the elevated treatment. The table also shows arbitrarily assigned protein spot numbers, putative identifications (description) and their respective accession numbers based on homology to a Mollusca nr database, spectral counts, log(e) values, isoelectric points (pI) and molecular weights (MW). aCO_2_ + eT: ambient CO_2_ and elevated temperature; eCO_2_ + aT: elevated CO_2_ and ambient temperature; and eCO_2_ + eT: elevated CO_2_ and elevated temperature. (XLSX 18 kb)
